# Supplementary material for: The complete mitochondrial genome of Gyps coprotheres (Aves, Accipitridae, Accipitriformes): phylogenetic analysis of mitogenome among raptors
Source: PeerJ. 2020 Nov 11;8:e10034. doi: 10.7717/peerj.10034 (PMC7666543; doi:10.7717/peerj.10034)
Supplement: Supplemental Information 1 [file peerj-08-10034-s001.docx]

>Cape vulture (*Gyps coprotheres*) mitochondrial genome

ataagtagat gagctatcta caaagcccta aacaagtaac aatataagta gatgagctat

ctacacgtcc tcgtagctta cagtaaagca tggcactgaa gatgccaaga tggctgccgc

atatagccca gggacaaaag acttagtcct aaccttactg ttaattcatg ctaaacatat

acatgcaagt atccgcgccc cagtgtaaat gccctcgatc tcttattaag atgacaggag

caggcatcag gtacacatca atcgtagccc aagacgcctt gctcagccac accctcacgg

gtactcagca gtaattaaca ttaagcaata agtgcaaact tgacttagtt atagcatatc

tccagggttg gtaaatcttg tgccagccac cgcggtcata caagaaaccc aagttaacag

tgtccggcgt aaagagtggg tgcttactat ccaagcggtt aggattaaac tgcagctgag

ctgtcataag cccaagatgt atctaaaacc accaccaaga cgatcctagc gcccacgacc

aactaaaccc cacgaaagcc agggcacaaa ctgggattag ataccccact atgcctggcc

ctaaatttcg atacttaccc caccaaagta tccgcctgag aactacgagc acaaacgctt

aaaactctaa ggacttggcg gtgccccaaa cccacctaga ggagcctgtt ctataatcga

taacccacgt tacaccctac caccccttgc ccaagcagcc tacataccgc cgtcgccagc

tcacctcttc tgaaaggcca atagtgagcc taatagcccg cccgctagaa agacaggtca

aggtatagct cacggagtgg aagaaatggg ctacattttc taaagtagaa gatctcacga

aaaggggtgt gaaacagccc ctggaaggcg gatttagcag taaatcggga caatagagcc

ctatttaaac cggccctgag gcacgtacat accgcccgtc accctcctcg caggctaccc

ttcccataat taataaccca atcagccgaa gatgaggtaa gtcgtaacaa ggtaagtgta

ccggaaggtg cacttagcat accaggacgt agctataata caaagcattc agcttacacc

tgaaagatac ctgctactta ctgggtcgtc ctgaagccaa actctagccc aaccacataa

caaccaacag ccaaaaagcc actttaccca caaactaaaa cattctccta acttagtata

ggtgatagaa aagttcccca ccgcccggcg cgatagagat ttcttgtacc gtaagggaaa

gatgaaataa tagtgaaagc ccaagcaaca cgcagcaaag atgaaccctt gtacctcttg

cattatggtt tagcaagaat aaccaagcaa aacgaattta agcttgccct cccgaaacct

aagcgagcta cttgtaggca gctgtctcag agcgaacccg tctctgttgc aaaagagtgg

gatgacctac tagtagaggt gaaaagccaa tcgagctagg tgatagctgg ttgcctgtga

gacgaatctt agttcctcct taattcacct ccacggacct tcagctaagc ccctacgtag

tgaatcaaga gcaatttaaa ggaggtacag ctcctttaaa aaaggataca acctcccgca

gtggataaag tttacttcct ccccaacctg taggccttca agcagccacc aacaaagagt

gcgtcaaagc tcctccccca agatacaaaa acaatacgac tccctcccca ctaacaggcc

aacctatcat aataggagaa ttaatgctag aatgagtaac tagggatacc cctcttaagc

gcaaacttac attagcacat tattaacagc gtctaattaa tgccacaatc tcaacaagct

agtacattaa aaccactctg ttagcccaac ccaggagcgc ctactagaaa gactaaaatc

tgcaaaagga actaggcaaa cccaaggccc gactgtttac caaaaacata gccttcagca

aacccagtat tgaaggtgat gcctgcccag tgaccctcag ttcaacggcc gcggtatcct

aaccgtgcga aggtagcgca atcaattgtc ccataaatcg agacttgtat gaatggctaa

acgaggtctt aactgtctct tgcagatagt cagtgaaatt gatcttcccg tgcaaaagca

ggaataagca cataagacga gaagaccctg tggaacttaa aaatcagcag ccaccacaca

taaatatgta cctaccaggc tcacagttaa aattaagcgc tggcttgcat ttttcggttg

gggcgacctt ggagaaaaga aaaacctcca aaaataagac catccctctt aactaagagc

gacccctcaa cgtacaaaca gtaaccagac ccaatagcaa ttgatcaatg gaccaagcta

ccccagggat aacagcgcaa tctctcccaa gagcccctat cgacggagag gtttacgacc

tcgatgttgg atcaggacat cctaatggtg cagccgctat taagggttcg tttgttcaac

gattaacagt cctacgtgat ctgagttcag accggagcaa tccaggtcgg tttctatcta

tgcagcactt tccccagtac gaaaggaccg gaaaagtaag gccaatacca caagcacgcc

ttctccccaa ataatgactc caactgaatc ataaagggaa ctccctacaa atccccaagc

cctagagaag ggaccgctag cgtggcagag cccggtaaat gcaaaaggct taagcccttt

atccagaggt tcaaatcctc tccctagccc catctacaat gactcgcccc tccaccttaa

ctcatcttat tataatccta tcctacgcaa tcccaatcct aattgccgta gccttcctaa

cactagtaga acgaaaagtc ttaagctata tgcaggctcg aaaaggccca aacattgtag

ggccattcgg actattacag ccagtagccg acggtgtaaa actattcatc aaagagcctg

tccgcccatc cacctcctct ccacttctct tcactataat acccatacta gccctccttc

tagctctaac catctgaatc cctctccccc tccccttctc ccttgccgac ttaaacctag

gcctactctt ccttctagcc atatcaagcc tagcagtgta ctcaatccta tgatctgggt

gggcctcaaa ctcaaaatat gcactaattg gggccctccg agcagtagca cagaccatct

cctacgaagt tacattagcc atcatcctcc tatccgtaat cctcctaagt ggtaactata

ccctacatgc actcaccatc actcaagaac ccctttatct catcttctcc tcctggcccc

tcacaatgat atgatatatc tccacactag ccgaaacaaa tcgagccccc tttgacctta

cagaggggga atcagaacta gtctcaggct tcaacgtaga atatgccgca gggccattcg

ccctattctt cctagctgaa tacgcaaaca ttatactaat aaacacacta actgcaactc

tgttcctaaa ccccagctca ctcaacctac cccctcaact attcacaata gccctggcaa

caaaagtctt gctcctctcc tccggattct tatgaattcg tgcctcctac ccacgtttcc

gctatgatca actcatacac ctcctctgaa aaaactttct acccttaacg ctagcactat

gcctctggca cattagcata ccaacctgtt atgcaggggt acctccttgc ttaaggaaat

gtgcctgaat gcaaagggcc actatgataa agtgaacata gaggtgtacc aaccctctca

tttcctaagt cttagcacct tagaaaagta ggaatcgaac ctacacagaa gagatcaaaa

ctctccatac ttcctttata ttatttccta gcagggtcag ctaacaaagc tatcgggccc

ataccccgaa aatgatggtt taaacccttc ccctgctaat gaacccccat acaaaactaa

tttcctcctt aagcctaatc ctaggcacaa ccatcacaat ctcaagcact cactgaataa

tagcctgagc tggactagaa ctcaacaccc tcgccatcat cccgttcatt tcaaaatccc

accaccctcg agctgttgaa gccacagtca aatattttct agtacaagca acagcctccg

ccctcctcct attctcaagc ataacaaacg catgatccac aggccaatga gacatcaccc

agctaaccca tcctacctcc tccctactgt taacagtagc aattgcaata aaactaggac

tagtgccttt ccacttctga ttcccagaag tactgcaagg ctcatcctta accactgccc

tacttctatc cacaataata aaattccccc caatcgttct cctctccctg acctctcatt

cactcaaccc aacactacta accactatag ccatcacctc agtagccctc ggagggtgaa

taggcctaaa ccaaacacag ctccgaaaaa tcctggcctt ctcatccatc gcccatctag

gatgaatagc cgcgatcatc atctacagcc ccagcctcac cctactaacc ttctacctat

acgtcctaat aaccaccact gtattcctca ctctcaactc aaccaaagcc ctaaaactag

tgacaataat aacatcatga acaaaatccc ccacactaaa cgcaactctc atactagtcc

tactctccct ggcaggactt cctccactaa caggcttcct ccccaaatga ctcatcatcc

aagaactaac caaacaagaa atggccacaa cagctacaat cattgccacc ctctccctcc

taggactatt cttctacctc cgcctcgcat accactcaac aattacacta ccccccaact

ccacaaatta cataaaacag tgacacttca ccaaatcaac aagcatcaca accgccatcc

tcgctaccct gtcaacgcta atcctgccgc tctcccccct aatcctgacc ctcaccgcca

cctagaaact taggataacc actaaaccga aggccttcaa agccttaaat aagagttaaa

ccctcttagt ttctgctaag atccgcagga cactatcctg catcctctga atgcaaccca

5221 gatactttaa ttaagctagg accttatcta gatagatggg cctcgatccc ataatatcct

agttaacagc taggtgcccc aaccagcagg cttctaccta ccagaccctg gcacatctct

aacgtgcatc aatgagtttg caactcacca tgaacttcac cacagggccg ataagaaggg

gaattaaacc cctgtaaaaa ggactacagc ctaacgctta gacactcagc catcttacct

gtgacatcaa tcaatcgatg actattctca accaaccaca aagacattgg cactctatac

ctaatcttcg gcgcttgagc tggtatagtc ggcactgctc tcagccttct tatccgtgca

gaactcggtc aaccaggcac tctcctaggc gatgaccaaa tctacaacgt agtcgtcacc

gcccacgcct ttgtaataat cttcttcata gttataccaa ttataattgg aggcttcgga

aactgactcg ttccacttat aattggtgct cccgacatag cctttccacg cataaacaac

ataagcttct gactactccc tccatccttc cttctcctac tagcctcctc aacagtagaa

gcaggagctg gtacaggatg aaccgtctac cccccactag ccggcaacat agcccatgct

ggagcctcag tagacttagc catcttctcc ctacacctag caggaatctc atccattcta

ggggcaatca acttcatcac cacagccatt aacataaaac cacctgccct ctcccaatac

caaacacccc tattcgtatg gtctgtcctc attaccgcag tcctactact actctcactc

ccagtcttag ccgctggcat cactatacta cttacagacc gaaacctcaa cacaacgttc

tttgaccccg ccggaggagg tgacccagtt ctataccaac acctcttctg attcttcgga

catcctgaag tctatatcct aattctaccg ggctttggaa tcatctctca cgtagtaaca

tactacgcag gtaaaaaaga accattcggc tacataggaa tagtctgagc catactatcc

attggattcc taggcttcat cgtatgggcc caccacatat tcacagtagg gatagacgta

gacacccgag catactttac atccgccact ataatcattg ccatcccaac cggtattaaa

gtcttcagct gactggcaac cctccatgga ggaactatca aatgggaccc cccaatacta

tgggccctgg gcttcatctt cctcttcact atcggaggtc taacagggat tgtcttagca

aactcttcac tagacattgc cctacacgac acatactacg tagttgctca cttccactat

gtcctttcaa taggagctgt ctttgccatt ctagcaggat ttacccactg attcccccta

ttaacaggat tcaccctaca tcctacgtga gccaaagcac atttcggagt tatattcaca

ggcgtaaacc taactttctt cccacagcac ttcctaggcc ttgccggaat gccccgacga

tactcagact acccagacgc ctacacccta tgaaatacca tatcctctat cggctcatta

atctcaataa cagccgtaat tatactaata ttcattattt gagaagcctt cgcttcaaaa

cgaaaagtcc tacaaccaga actaaccatg actaacgtcg aatgaatcca cggctgccca

cccccatacc ataccttcga agagccagcc tttgtccaag tacaagaaag gaaggaatcg

aacctccgta cactggtttc aagccagccg catctaacca cctatgcttc tttctttaat

gggatgttag taaaccaatt acataacctt gtcaaggtta aatcacaggt aaaagccctg

tacaccccac catggctaac cactcacagc tcggattcca agacgcctca tcccctatca

tagaagaact cgtcgaattc cacgaccacg ccctaatagt tgcactagcc atctgcagcc

tagtcctata ccttctcgca ctcatattaa tagaaaaact atcctcaaac actgtagatg

ctcaagaaat tgaactaatc tgaacaatcc taccagccat cgtcctcatc atactcgccc

ttccatccct acaaatccta tatataatag acgaaatcaa cgaaccagac ctcacactaa

aggccatcgg ccatcaatgg tactgaacct acgaatacac ggacttcaaa gacctgacat

tcgactccta catagttcca acaacagacc tcctaccagg acacttccga ctcctagaag

ttgaccaccg cataatcgtc ccaatagaat cccccatccg cattatcgtc acagccaatg

acgtacttca cgcctgagca gtccctgcgc taggtgtaaa aactgatgca atcccaggac

gactaaacca aacatcattt atcaccaccc gacctggggt gttctacggc caatgctcag

aaatctgcgg agccaatcac agctatatgc caatcgtagt agaatcaacc cccctagccc

acttcgaaca ctgatcctca ctcatatcct cctaatcatt aagaagctat gtaacagcac

tagcctttta agctagagaa agaggactac ctacccctcc ttaatgacat gccacaactt

aacccagccc catggctcta tatcatacta gcatcatgat taatccttgc actagtcatc

caacccaagc tcttaccctt cacctcaact aactccctct ttaacaaaac tactataact

gccaacaccc cctcctgaaa ctgaccatga atctaagctt cttcgaccaa tttacaagcc

cctgcttcct aggtatccca ctcatcctaa tttcaatact attccctact ctactattcc

cctcacccac taaccgatga gtcactaatc gcctctctac cctccaatta tgactcattc

atcttatcac aaaacaacta atattgccct taagtaaagc aggacataaa tgggccctaa

tcttaacatc actaataata ctcctactta caatcaactt actaggacta ctgccctaca

cattcacccc gactacccaa ctatcaataa acatagccct tgccttccct ctttgactag

ccacactcct aacaggacta cgaaaccaac ctactacatc cctaggccat ctcctacctg

aaggcacccc aacacctcta atccccgccc taatcctaat cgaaactacc agcctactta

tccgtcccct agccttagga gtccgcctca cagcaaacct aacagcaggc caccttctaa

tccaacttat ctctaccgcc actaccgccc tactccccat cctcccagca gtatctatcc

taaccatact aatcctgcta ctactaacca tcctagaaat cgcagtagcc ataatccaag

cctacgtctt cgtcctactc ctaagcctat atctacaaga aaacatctaa tggctcacca

agcacactcc taccacatag tagaccccag cccttggcct attttcggcg cagcagctgc

cttactcact acctcaggtc taatcatatg attccactac aactcctccc aactcttatc

cttaggccta ctctccatac ttctagttat attacaatga tgacgagata ttgtacgaga

aagtacattc caaggccatc atactcctac tgtccaaaaa ggtctgcgat acggaataat

cctattcatt acatccgaag cattcttctt ccttggcttc ttctgagcat tcttccactc

tagcctagct cccaccccag agctaggcgg acagtgaccc ccaacaggaa tcaatcccct

caaccccctt gaagtacccc tacttaacac agcaatcctc cttgcctccg gtgtcaccgt

aacctgagca caccacagca tcatagagag caaccgaaaa caagcaattc acgcactcac

cctaaccatc ctcttgggct tctacttcac agccctacaa gcaatagagt actacgaagc

tccattctca attgctgacg gcgtatacgg ctcaaccttc tttgttgcta caggttttca

cggactccat gtaatcattg ggtcatcctt cctatcagtc tgcctcctac gactaattaa

cttccacttc acatctaacc atcatttcgg attcgaagca gctgcctgat actgacactt

cgtagacgtc atctgacttt tcctctacat aaccatttac tgatgaggat cttgctcttc

tagtatatta attacaattg acttccaatc tataaaatct ggtataaccc cagagaagag

caattaatat agtcaccttt acacttgccc tatccctcac cctaagcacc atcctaatca

tattaaacct ctgacttgcc caaacaagcc cagactcaga aaaactatca ccatacgaat

gtggcttcga tcctctaggc tctgcccgac taccattctc catccgattc ttcctcagta

gctatcctat tcttactatt cgacctagaa atcgcacttt tactaccact accatgagcc

atccagctcc aatcccccat ctccactcta gcctgagctt ccgttattat ctctctcctc

acattaggat taatctatga atgactccaa ggggggctag aatgagccga ataaacatag

aaaggtagtc taactaagac agttgatttc gactcaacaa atcatagatc agccctatgc

ctctctccat gtcacttcta catctaagct tctactcttc attcacctta agctgcttag

gcctagcctt ccaccgaact cacttactct ccgccctact atgcctagag agcataatac

tttccatata cattacccta tctgtctgac ctgtagaaac ccaaataaca tctatcaccc

tagctcctgt attcatacta acattctcag cctgtgaagc aggcactggc ctagctatgc

tagttgcctc cacgcgaact cacggctctg accacctaca caacctaaac ctcctgcaat

gctaaaaatc ctcctcccaa cagctatact cctacctacc gccctcctat cccctcaaaa

attcctgtga actaacacta ccacccacag cctcctaatt gccaccatta gtctccactg

attacttccc acatattatc cctacaaaaa cctcactcaa tgaacatgca ttgatcaaat

ctcatccccc ctactagtcc tatcctgctg actactccca ctcataatct tagcaagcca

aaaccaccta caacacgaac cgccagcacg caaacgaatc ttcatcacta ccctaatcac

agtacagccc ctacttcttc tagcattctc agccaccgaa ctaatactat tctacattac

attcgaagca accttaatcc ccaccctagt cttgatcaca cgctgaggaa gccagccaga

acgcctaagc gcaggcattt atctactatt ctacacctta atcagctccc tgcccctact

agtcacaatc ctgtatctcc acacccacat cggcaccctt caccttacaa tactcaaact

atcccacccc tcacttacca cctcatgaac tgatctcctc ctaagcctag cgctattaat

agccttcata gtaaaagccc ccctctatgg cctccaccta tgactgccca aagcccacgt

agaagcccca attgcagggt ccatattact cgctgctcta ctcctaaaac taggaggtta

tggcatcata cgcctcaccc tcctaattgg ccctctctca gcccacctac actacccctt

cctcaccttg gccctctgag gcgcgctaat aactagctcg atctgcctgc gtcaaactga

cttaaagtcc ctcattgcct actcctccgt aagccacatg ggcttagtca tcgctgcaag

catcatccaa acccactgat ccttctcagg ggcaatactc ctaataatct ctcacggcct

tacctcctca atactattct gcttagccaa tacaacctac gaacgcaccc acagccgaat

ccttctcctg acacgaggcc tacaacccct cctgcccctt atagctacct gatgactact

agccaacctc acaaacatgg ctctaccacc aaccaccaac ctgatagcag aactaaccat

cataaccgca ctattcaact gatccacccc caccatcatc ctcacagggg ccgcaaccct

actaactgct tcatacaccc tattcatgct actaataacc caacgaggaa cactaccaac

ccacatcaca tccctacaaa actcaagcac acgagaacat ctcctaataa cccttcacat

cgtcccccta ctcctcctaa tcctcaaacc agagacaatc tcaggaatac ccttatgcag

atatagtttc aacccaaaca ttagattgtg attctaaaaa tagaagttaa acccttctta

cctgccgagg gagagttcaa ccagcaagaa ctgctaatcc ttgcatctga gtttaaaacc

tcagtcccct taacttttaa aggataacag taatccactg gtcttaggag ccactcatct

tggtgcaaat ccaagtaaaa gtaatggaaa tgatactact cctcaacacc tccatacttc

ttacactagc aattctcctc ataccaattc tcctcccact actcaccacc cactcaacct

ccccaaccac catcacacgc actgtcaaaa ttgccttcct aaccagcttg acaccaatat

gccttttcat atactcagga tcagaaagta ttgtctctca ctgagaatga aaattcatta

taaacttcaa aatccccatc agctttaaaa ttgaccaata ctcaataatc ttctttccca

ttgcactatt cgtaacatgg tccattctcc aatttgcatc atgatatata gcatcagagc

catatatcac aaaattcttc tccttcctct taacattcct aatcgctatg cttacactaa

caattgccaa caacatattc cttcttttta ttggttgaga aggagtcgga atcatatcct

tcctactaat cggatgatga caaggccgcg cagaggccaa cacagctgca ctccaagctg

taatctacaa ccggatcgga gacatcggcc tcatcctaag catagcatac ctagcctcaa

caacaaacac atgagaaatc caacaaacct tctcccccag ccaaacccca accctccccc

tactaggact cattctagct gctacaggaa aatctgccca attcggcctt cacccatgac

tacccgctgc tatagagggc ccaaccccag tttctgccct actccactcc agcaccatag

tagtagcagg aatctttcta ctcatccgca cccaccccat acttgcaacc aaccaaatcg

ccctcacttc atgtttgtgc ttaggggcac tatccacatt atttgcggcc acctgcgccc

taacacaaaa tgacatcaaa aaaatcattg ctttttccac atcaagccag cttggattga

tgatagttac catcggacta aaccttcccc aactagcctt cttccacatc tcaacacatg

cattcttcaa ggccatgcta ttcctctgct cagggtcaat catccatgcc ctcaatggag

agcaagacat tcgaaaaata ggaggccttc aaaaactact cccaacaact accacctgcc

taaccatcgg aaacttagcc ctaatgggaa cgccattttt agcagggttc tactcaaaag

acctaatcat tgaaagctta aacacctcct atctaaatgc ttgagcttta cttctaacac

tcctagcaac ctcctttaca gcaacgtata gcctacgcat aaccctacta gttcaaacag

aattcccccg catgcccaca atcacaccca taaatgaaaa caacccaaca ctcatcaacc

caatcacccg actcgccata ggcagcatca cagccggcct actcatcaca tcctacattc

ctcccacaaa aacacctcca ataaccatgc ccacactcac aaaaaccaca gcaattgtca

tcacagccct aggacttatc ctagccctag agctttcaaa cataacacac accctaactc

aaccaaaaca aaccacatac ataaacttct cctccatatt aggatacttc aactccctaa

cacatcgcct cagctcccta accctactaa acaacggaca aaaaattgcc tcccacctaa

tcgacctctc ctgatacaaa aaaataggcc cagaaggaat cgctgatctc caactcacaa

taactaaaac ctcaacctcc ttccacaccg gactactcaa agcctactta gggaccttcg

ccctatcaac cctcatcatc ctcctatcag cacattaacc ccctaaacca atggccccca

acccacgaaa atcccacccc ctactaaaaa taatcaacaa ctccctaatc gacctaccta

cccctcccaa catctccatt tgatgaaact tcgggtctct tctaggaatc tgcctactga

cacaaatctt gaccggcctc ctacttgcaa tacactacac cgcagacaca tccttagcct

tttcatccgt tgcccacaca tgccgaaacg tacagtacgg gtgactaatc cgcaacctac

atgccaacgg agcatccttc ttcttcattt gcatctacct acacatcggc cgaggtctct

actacggctc atacctatat aaagagacct gaaacacagg aattatcctc ttgctcaccc

tcatggcaac tgccttcgta ggttacgtcc taccatgagg acaaatatcc ttctgagggg

ctacagtcat caccaaccta ttctcagcca tcccatacat cggacaaacc cttgtagaat

gagcctgagg aggcttctcc gtagataacc ccaccctcac ccgattcttc gccctacatt

tcttacttcc atttgtaatc gcaggtctta ctctaatcca ccttaccttc ctccacgaat

ctggctcaaa caacccccta ggcattatct caaactgcga caaaatccca ttccacccat

acttctccct caaggacatt ctagggttcg tcctaatact actcccatta acagccctag

ccctattctc cccaaaccta ctgggcgacc cagaaaactt caccccagca aacccactag

tcacaccccc acatattaag ccagaatgat attttctatt cgcatacgct attctacgct

caatcccaaa taaactagga ggagtactag ccttagctgc ctccgtacta atcctattcc

taatcccctt tctacacaaa tccaagcagc gcacaataac ctttcgacct ctctcccaac

tcctatactg aaccctaatc gccaatctcc tcatcctcac atgaatcggc agccagccag

tagaacaccc cttcatcatc atcggccaac tagcctccat cgcctacttt actatccttc

tagttctctt ccccttaact ggggccctag aaaataaaat gctcaaccac taaatactct

aatagtttat aaaaacattg gtcttgtaaa ccaaagactg agggttatcc ccctcttaga

gttctcggcc caaaagggcc attattgcca aattttaact aaaaaccacc ctagttaagg

ccccccccct acccccccca tgtatcattg tacacccatg tatcattgta cattacacta

tttgccccat attatgcacc atgctatgat atacatatta atgcatgtac tatatacata

cttatgtatt acgggcatac tactatctag gccatctcta cattcaggga tcagttatca

ctccatgaac gactgattag aacttcatgc tcggccggaa tgttcctaaa tctcccctcg

gactagaggc ataccaaagg ttgcttgtct gcgtattagt agtttttccc tacggatatt

cttgaggact aacgattcat ggtagcaggt catatggcgt aactttgttt aatcctctaa

tatcgccctt tcttgtagta ccggcgtctc ggaccaggtt atctattaat tggtcttcct

ccgagaaatc agcaaccgac tgcacatggg atcctacgtt actagcttca ggatcttact

ttccccctgc cccggagctc ttcttgctct tttgcgcctc tggttcctat atcaaggcca

caacttggtt gatcctatga tcttgctctt tacgaataca tctggtatgg ccttcggccg

taaggtcatc attctccctc ttaatcgcgg caaccgggtg gctctttact attggttccc

tttttttttt ggggtaactt cacaggttgc cctcatatac gcatcgcggc gcatacaatc

tcaggacatg gacgctgctg gctctcggcc ggtgtttgga tctcaagagt tgattaatga

gacggttggc gtattgcggt aatccaccga taacactgat gcactgtcag ggccatttgg

ctatggcgtg tccacggact ttacccatgg tgctatttag tgaatgcttg tgggacatgt

ttttctattt ttcctttcct cttactttct atactacact aggcacattc gggttaaaac

gctaaccgtg tttttttaca aattttatca aatttttttc actttttttc atccgtcgaa

ggcactggaa ttccattaat aaatcaaatc gttatcatta atttgtttca tcatcattca

ccattttttc cacgaactca acccacataa cctctaaaat tccatctata acaacccatt

aacaaacttc agaaaaaaag ggctcaaacc tctatcacca gctcccaaag ctggtatttt

acattaaact attctctgat tttctctttc cttccccccc taaactgccc gaatcgcccc

acgagacatc cctcgcacaa gctccaacac aacaaacaat gtcaacaaca atccccatcc

tgccactaaa aacatccctg ccccccacga ataaaacata gcaacaccac taaaatccaa

ccgagctaca aaagtaccac ctccgtcaac agtgactact ccggccttcc aacattcaac

ctcactagca accaccccta caacgagcac caagatcatc cctaccccat accctaaaac

ccgccaatca cctcaagcct ctggaaataa atccgctgcc aagcagaccg aatagacaaa

aaccactaac atcccaccca agtacaccat aaacaatacc agagctacaa acggtactcc

caaacttacc aaccacccac accccgcaac agaccctagc accaacccaa ccaccccata

ataaggtgat gggtttgatg caactgctag ccctcccaaa acaaaccctg cgcccaaaag

aaggacaaaa taggccataa tttctgcttg gcttctctcc aaaatctacg acctgaaaaa

tcgtcgttgt aaacttcaac tacagaaacc aacataaacc acctcaccct cagatcttct

tttttcattc ttttctactt cttttccatt cttttttcat tctttttc
