# Supplementary figures and images for: The complete mitochondrial genome of Gyps coprotheres (Aves, Accipitridae, Accipitriformes): phylogenetic analysis of mitogenome among raptors
Source: PeerJ. 2020 Nov 11;8:e10034. doi: 10.7717/peerj.10034 (PMC7666543; doi:10.7717/peerj.10034)

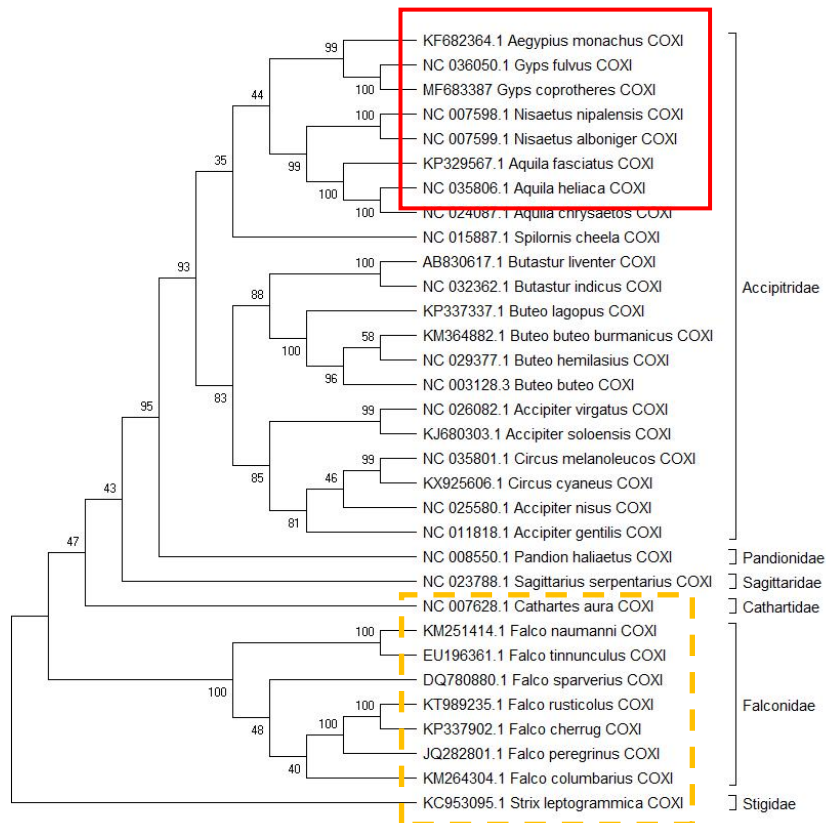

Supplement: Supplemental Information 2 — Results of Phylogenetic analyses using maximum likelihood (ML) analysis indicated evolutionary relationships among 32 raptor species based on COXI sequences. Strix leptogrammica was used as outgroup. Bootstrap support values for ML analyses are indicated on the nodes. The solid border rectangle indicates a close phylogenetic relationship between old world vultures and eagles confirmed with high bootstrap values with greater chances of shared susceptibility based on their close evolutionary relationship. While distantly related raptor bird species included within the broken border rectangle are less likely to succumb to diclofenac toxicity as reported for turkey vulture (Cathartes aura) (Rattner et al., 2008). [file peerj-08-10034-s002.pdf]
